# Association of autoimmune comorbidities in persons with multiple sclerosis from a population-based study with genetic linkage

**DOI:** 10.1177/20552173251349671

**Published:** 2025-07-03

**Authors:** Roberto Gnavi, Nadia Barizzone, Roberta Picariello, Paolo Emilio Alboini, Nicola Pomella, Muralidharan Thavamani, Martina Tosi, Endri Visha, Valentina Ciampana, Domizia Vecchio, Paola Cavalla, Maurizio Leone, Sandra D'Alfonso

**Affiliations:** 159147 Servizio Sovrazonale di Epidemiologia ASL TO3, Regione Piemonte, Collegno (TO), Italy; Department of Health Sciences, 60252 University of Eastern Piedmont, Novara, Italy; 429308 Servizio Sovrazonale di Epidemiologia ASL TO3, Regione Piemonte, Collegno (TO), Italy; Fondazione IRCCS Casa Sollievo della Sofferenza, San Giovanni Rotondo, Italy; Department of Health Sciences, University of Eastern Piedmont, Novara, Italy; Department of Translational Medicine, Neurology Unit, 9256 Maggiore Della Carità Hospital, University of Eastern Piedmont, Novara, Italy; Department of Neuroscience and Mental Health, AOU Città della Salute e della Scienza di Torino University Hospital, Torino, Italy; Scientific Direction, Fondazione IRCCS Casa Sollievo della Sofferenza, San Giovanni Rotondo, Italy; Department of Health Sciences, University of Eastern Piedmont, Novara, Italy Center on Autoimmune and Allergic Diseases (CAAD), Novara, Italy

**Keywords:** Multiple sclerosis, autoimmune disease, comorbidity, shared genetics, electronic health archives, population-based study, polygenic risk score

## Abstract

**Background:**

Comorbidities are a critical concern for clinicians in both the treatment and diagnosis of multiple sclerosis. Autoimmune diseases, including multiple sclerosis, often co-occur within individuals. However, most studies examining the incidence or prevalence of autoimmune diseases in persons with multiple sclerosis compared to healthy controls have used relatively small sample sets, with only a few being population-based.

**Objectives:**

To analyze the co-occurrence of other autoimmune diseases in persons with multiple sclerosis and determine whether common genetic susceptibility factors contribute to the co-occurrence of autoimmune diseases.

**Methods:**

We conducted a population-based study using administrative health records to include all residents of Piedmont, an Italian Region with about 4.3 million inhabitants, identifying individuals with multiple sclerosis and 14 other autoimmune diseases. For a subset of persons with multiple sclerosis with available genome-wide genotyping data, we investigated the influence of their genetic backgrounds using a polygenic risk score.

**Results:**

The prevalence of all 14 tested autoimmune diseases was higher in persons with multiple sclerosis compared to those without multiple sclerosis. Furthermore, persons with multiple sclerosis with autoimmune disease comorbidities had a higher polygenic risk score compared to persons with multiple sclerosis without comorbidities.

**Conclusion:**

Our findings confirm the co-occurrence of multiple sclerosis with several autoimmune diseases, and suggest that shared genetic susceptibility factors may influence this association.

## Introduction

In persons with multiple sclerosis (pwMS), the presence of comorbidities has been associated with a diminished quality of life, increased risk of relapse, accelerated disease progression, and higher severity of the disease, as measured by the Multiple Sclerosis Severity Score (MSSS).^1,2^

Common comorbidities in pwMS include depression (23%), anxiety (22%), hypertension (19%), and chronic pulmonary diseases (10%), reflecting the higher prevalence of these conditions in the general population.^3^ Comorbidities can complicate the management of multiple sclerosis (MS), particularly when they present before or early in the disease course, leading to delays in diagnosis and challenges in providing timely and effective treatment, underscoring the importance of a comprehensive assessment of comorbidities in pwMS.

This aspect becomes particularly relevant when we consider that individuals who have one autoimmune disease (AID) are more likely to develop additional AIDs compared to the general population, as highlighted in a recent population-based study of 19 common AIDs in the UK population.^4^ One possible explanation for this co-occurrence is that common genetic susceptibility factors for autoimmunity interact with other genetic and environmental determinants, leading to the manifestation of multiple autoimmune conditions.^5,6^ In this regard, several cohort and cross-sectional studies suggest that MS is associated with a higher risk of other AIDs, both in MS patients and their relatives.^7,8^ For instance, a recent systematic review on comorbidity in individuals with MS has shown the coexistence of several immune-mediated and inflammatory diseases, including psoriasis, asthma, type 1 diabetes (T1DM), autoimmune thyroiditis, celiac disease, Sjogren's syndrome, inflammatory bowel disease (IBD), rheumatoid arthritis (RA), systemic lupus erythematosus (SLE), and atopic dermatitis. However, the reported prevalence ratios were highly variable across studies,^1^ likely influenced by differences in study design, sample size, and demographics. Interestingly, while many studies based on relatively small sample sets—few of which are population-based—report an increased co-occurrence of AIDs in pwMS, a large population-based study in the UK observed a notably low co-occurrence rate and even inverse associations for certain autoimmune conditions.^4^ Such discrepancies may be ascribable to differences in geographical, genetic, environmental, and ethnic factors of the studied populations, underscoring the need for conducting further analyses in more homogeneous settings.

To address these inconsistencies, we have examined the co-occurrence of 14 AIDs in pwMS using data from population-based, routinely collected electronic health archives (EHAs), covering the entire population of Piedmont, a region in northwestern Italy. Furthermore, we have evaluated the feasibility of linking genetic data with EHA to determine whether a higher genetic burden of autoimmune-associated variants correlates with increased autoimmune comorbidity.

## Methods

### Study population and data sources

The study was conducted in Piedmont, a region in northwestern Italy with ∼4.3 million residents, representing 7.5% of the Italian population. The study comprised two distinct phases. In the first phase, we compared the frequency of 14 AIDs in individuals with or without MS. The choice of diseases selected for this study was dictated by the availability of specific codes in the linked administrative databases and of algorithms used in prior studies. In the second phase, we analyzed a subset of pwMS for whom genome-wide genetic data were available, enabling a more detailed genetic analysis. For this group, we calculated a polygenic risk score (PRS) using known susceptibility variants from the literature and used it to compare pwMS with autoimmune comorbidity (i.e. at least one AID) and those without autoimmune comorbidity.

The study was approved by the Ethics Committee of “Fondazione IRCCS Casa Sollievo della Sofferenza.”

### Identification of people with AIDs from public health databases

In Italy, all residents are covered by a universal public health system that provides comprehensive healthcare services. Data from these services are routinely collected in an automated system of linked databases, with all personal information pseudo-anonymized. These databases are enriched with a unique anonymous identifier, encrypted to protect patient privacy, allowing linkage across different databases.

Four EHAs were employed to identify pwMS in the period from 1 January 2012 to 31 December 2017^9^: hospital discharges occurring anywhere in Italy, drug prescriptions carried out by public and private pharmacies, certifications of chronic diseases for exemption from co-payment of drugs, and a list of persons resident in long-term care facilities. Using these data sources and a previously validated algorithm with high reliability (sensitivity 95.9% and specificity 99.9%),^9^ we identified pwMS residing in Piedmont as of 31 December 2017, without any age or sex exclusion. Residents without MS at the same date, and within the same age range of pwMS, were identified as the MS-free population.

To identify comorbidities with other AIDs, these two populations were linked to the same previously described regional EHA over the past 5 years (i.e. from 2012 to 2017) using algorithms previously utilized in multiple studies.^10–14^ We successfully performed this linkage for 14 AIDs: RA, celiac disease, chronic inflammatory demyelinating polyneuropathy (CIDP), T1DM, Hashimoto thyroiditis, hypothyroidism (non-iatrogenic), SLE, myasthenia gravis, psoriatic arthritis, sarcoidosis, Behçet syndrome, Guillain-Barré syndrome (GBS), Sjogren's syndrome (SS), and ankylosing spondylitis (AS). Details of the algorithms used to identify patients affected with the above 14 AIDs are reported in Supplemental Table S1.

Unfortunately, we could not perform linkage for IBD, a relatively common immune-mediated disease, due to an error in the coding of IBD in the exemption from co-payment archives, which prevented its use.

For each subject, we retrieved demographic information, including age and sex. Since pwMS are more likely to be hospitalized, which may increase the probability of being diagnosed with autoimmune comorbidities compared to those without MS, we also accounted for the number of hospitalizations for any cause occurring anywhere in Italy in the 5 years prior to the study period (2013–2017) as a potential confounder.

### Sample set for genetic analysis

A subset of pwMS consisting of 637 individuals, including 65 with at least one of the aforementioned AIDs and 572 without comorbidities, had genome-wide genotyping data available. These individuals had been genotyped using either the Illumina Infinium Global Screening Array-24 v3.0 BeadChip or the Illumina Human 660 Quad platform (Illumina, Inc). Pre-imputation quality checks included the removal of individuals with sex discrepancies (between genetic and clinically reported data), duplicates (genetically identical by descent individuals), and removal of individuals with heterozygous rate > 3 standard deviation from the mean, removal of principal component (PC) outliers, and removal of individuals with a low genotyping rate. Single nucleotide polymorphisms (SNPs) with genotype rate < 90%, minor allele frequency < 0.01, and Hardy-Weinberg *p*-value < 1×10^–6^ were filtered out. The final dataset of 483,799 SNPs was then imputed against the Haplotype Reference Consortium panel, using the Michigan Imputation Server. As additional post-imputation quality control, we filtered out SNPs with *R*^2^ < 0.6 and minor allele frequency < 0.001, obtaining a final panel of 9,902,537 bona-fide imputed SNPs spanning the entire genome. The dosages were converted to best-guess genotypes.

### Statistical analysis

AIDs frequency was calculated by dividing the total number of individuals with each disease by the total number of pwMS and non-MS subjects residing in Piedmont who were alive on 31 December 2017. Population data were retrieved from the Unique Regional Archive of residents covered by the regional health system.

Differences in baseline characteristics between pwMS and the MS-free population were evaluated using either the χ2 test or the analysis of variance, as appropriate.

Logistic regression, adjusted for age (continuous), sex, and number of hospitalizations (continuous) was used to compare the frequency of AIDs between pwMS and individuals without MS. The results are presented as odds ratios (ORs), with 95% confidence intervals, for each AID. All analyses were performed using SAS System version 9.4.

### Polygenic risk scores

For each AID, including MS, we searched public databases (i.e. GWAS Atlas, GWAS Catalog, and Phenome scanner), as well as the broader literature, for genome-wide association studies (GWASs) to identify susceptibility variants. We defined a set of disease-associated variants exceeding the GWAS significance threshold (*p* < 5 × 10^–8^) for nine of these AIDs: MS, RA, celiac disease, T1DM, Hashimoto thyroiditis, hypothyroidism, SLE, psoriasis, and AS. The number of non-human leukocyte antigen (HLA) SNPs included in each disease-specific score ranged from 23 (AS) to 201 (MS) (Supplemental Table S2).

For each of these nine diseases, we generated a disease-specific PRS for a random subset of 637 pwMS with available GWAS genotyping data using in-house scripts. Each PRS was calculated as the unweighted sum of risk alleles for all non-HLA disease-associated variants.

Each disease-specific PRS was then normalized by dividing the absolute score of each pwMS by the maximum achievable score (2 × number of SNPs). The nine normalized scores were then summed to construct the cumulative PRS (*PRS_comorb*) for each individual.

To account for the effect of HLA variants, we complemented each disease-specific PRS with the burden of the HLA variants associated with each AID (Supplemental Table S3). The genotype at classical HLA alleles was inferred by genotype imputation, using the Michigan Imputation Server.^15^ We then repeated the normalization process and summed the nine normalized scores to construct a cumulative PRS (*PRS_comorb_HLA*).

To assess population stratification, accounting for genetic ancestry, we calculated PCs using Plink software and removed two outliers, defined as samples exceeding three standard deviations from the mean of any of the first three PCs. The plots of the first three PCs are reported in Supplemental Figure S1. We observed a general overlapping of MS samples with and without comorbidity.

To assess the statistical significance of the PRS differences between pwMS with and without AID comorbidity, we generated an ANCOVA model using age, sex, and the first three PCs as covariates.

## Results

As of 31 December 2017, administrative health database records allowed us to identify 8850 pwMS and 4,265,864 individuals without MS residing in Piedmont.

We compared the two populations, stratified by the presence of each analyzed AID, across several demographic characteristics (Table 1). Most AIDs were found to be more frequent among women, especially among pwMS, which is consistent with the known higher prevalence of these conditions in females. In particular, the frequency of women among pwMS was significantly higher for celiac disease, CIDP, T1DM, and hypothyroidism.

**Table 1.** Characteristics of the study populations with or without multiple sclerosis stratified by comorbidity status.

				PwMS *N* = 8850		Without MS *N* = 4,265,864				*p*-values	
Disease	*N*	% women	Mean age	Mean number of hospital discharges^a^	*N*	% women	Mean age	Mean number of hospital discharges^a^	Women	Age	Discharges
Rheumatoid arthritis	54	85.2	53.8	2.9	20,793	73.6	63.3	1.3	0.0535	<0.0001	<0.0001
Celiac disease	61	86.9	43.6	2.3	12,802	72.0	37.0	0.7	0.0097	0.0085	<0.0001
Chronic inflammatory demyelinating polyneuropathy	24	66.7	55.8	4.8	732	36.5	63.5	3.0	0.0026	0.0166	0.0162
Type 1 diabetes	77	67.5	49.3	3.1	16,692	45.0	53.1	1.7	<0.0001	0.1300	<0.0001
Hashimoto thiroiditis^b^	236	90.3	47.2	1.9	54,821	89.3	52.9	0.7	0.6217	<0.0001	<0.0001
Hypothyroidism	492	90.9	55.5	2.2	142,112	83.4	64.3	1.1	<0.0001	<0.0001	<0.0001
Systemic lupus erythematosus	15	100.0	49.6	3.5	3267	87.3	55.3	1.5	0.1403	0.1389	0.0036
Myasthenia gravis	10	80.0	52.0	3.5	1619	53.9	65.4	1.9	0.0990	0.0109	0.0600
Psoriatic arthritis	26	53.9	53.6	2.4	8560	50.6	58.1	1.0	0.7389	0.0941	<0.0001
Sarcoidosis	8	50.0	46.4	4.8	1648	52.2	53.7	1.6	0.9018	0.1326	<0.0001
Behçet syndrome	10	70.0	39.1	5.1	401	56.1	45.8	1.7	0.3815	0.1452	<0.0001
Guillain-Barré syndrome	14	64.3	52.9	4.1	1446	49.3	59.6	2.3	0.2646	0.1813	0.0116
Sjogren's syndrome	11	100.0	58.0	4.0	2662	93.6	64.7	1.4	0.3864	0.0934	0.0002
Ankylosing spondylitis	7	42.9	48.9	3.9	1977	42.5	52.4	1.1	0.9864	0.5020	0.0002

PwMS: persons with multiple sclerosis; MS: multiple sclerosis.

^a^ For any reason in the preceding 5 years.

^b^ Excluding cases treated with certain thyrotoxic drugs (e.g. interferon beta 1A, interferon beta 1B, peginterferon beta 1A, and alemtuzumab) in the 12 months prior to diagnosis.

When stratified by the presence of each AID, the mean age also differed between the two populations. Among pwMS, the mean age was lower for patients with RA, CIDP, Hashimoto thyroiditis, hypothyroidism, and myasthenia gravis, while it was higher for those with celiac disease. Across all AIDs, pwMS consistently had more previous hospitalizations compared to non-MS subjects, with the exception of those with myasthenia gravis.

Table 2 shows the association with the presence of the 14 AIDs in pwMS and people without MS, along with the results from the logistic regression models. As expected, the two most represented AIDs in both populations were hypothyroidism and Hashimoto thyroiditis, whereas all other AIDs affected fewer than 10/1000 individuals. All tested AIDs were more frequent among pwMS. In particular, CIDP, Behçet syndrome, and GBS showed the highest ORs.

**Table 2.** Association with the presence of the 14 AIDs with MS.

	Prevalence X 1000			
Disease	pwMS	Without MS	OR adjusted for age and gender^a^	OR adjusted for age, gender, and hospitalizations^a^
Rheumatoid arthritis	6.10	4.87	1.21 (0.92–1.58)	0.93 (0.71–1.22)
Celiac disease	6.89	3.00	2.29 (1.78–2.94)	2.01 (1.56–2.59)
Chronic inflammatory demyelinating polyneuropathy	2.71	0.17	19.38 (12.88–29.17)	13.53 (8.91–20.55)
Type 1 diabetes	8.70	3.91	2.32 (1.85–2.90)	1.54 (1.22–1.93)
Hashimoto thyroiditis	26.67	12.85	1.68 (1.48–1.92)	1.61 (1.41–1.83)
Hypothyroidism	55.59	33.31	1.59 (1.45–1.75)	1.32 (1.20–1.45)
Systemic lupus erythematosus	1.69	0.77	1.80 (1.09–3.00)	1.33 (0.79–2.23)
Myasthenia gravis	1.13	0.38	3.40 (1.82–6.33)	2.43 (1.29–4.59)
Psoriatic arthritis	2.94	2.01	1.51 (1.03–2.22)	1.29 (0.88–1.90)
Sarcoidosis	0.90	0.39	2.32 (1.16–4.65)	1.72 (0.85–3.48)
Behçet syndrome	1.13	0.09	11.79 (6.29–22.11)	9.13 (4.84–17.22)
Guillain-Barré syndrome	1.58	0.34	4.93 (2.91–8.35)	3.43 (2.00–5.87)
Sjogren's syndrome	1.24	0.62	1.77 (0.98–3.20)	1.36 (0.74–2.47)
Ankylosing spondylitis	0.79	0.46	1.80 (0.85–3.77)	1.48 (0.72–3.11)

AIDs: autoimmune diseases; MS: multiple sclerosis; PwMS: persons with multiple sclerosis.

^a^ Persons without MS represent the reference group for the odds ratios (OR), adjusted for age and gender, as well as for ORs adjusted for age, gender, and the number of previous hospitalizations.

95% confidence intervals are provided in brackets.

The inclusion of the number of hospitalizations as a covariate in the logistic model attenuated the strength of these associations, even though they remained statistically significant for the following conditions: CIDP (OR = 13.5), Behçet syndrome (OR = 9.13), myasthenia gravis (OR = 2.43), GBS (OR = 3.43), celiac disease (OR = 2.01), Hashimoto thyroiditis (OR = 1.61), T1DM (OR = 1.54), SLE (OR = 1.33), and hypothyroidism (OR = 1.32).

To assess the influence of the shared genetic backgrounds on autoimmune comorbidity in pwMS, we generated a PRS, labeled *PRS_comorb_HLA*, for a random subset of 637 pwMS with available genotyping data, considering the known HLA and non-HLA genetic susceptibility loci for nine AIDs, including MS, RA, celiac disease, T1DM, Hashimoto thyroiditis, hypothyroidism, SLE, psoriasis, and AS.

The subset of sampled patients consisted of 423 women and 214 men (sex ratio = 1.98:1), substantially overlapping with the distribution in the whole population study (5979 women and 2871 men, sex ratio = 2.08:1). The mean age was 52.0 years (SD 11.5) compared to 50.0 (SD 13.7, *p* < 0.0005) for the general MS population. The genetic subgroup consisted of 19 samples presenting with clinically isolated syndrome, 489 with relapsing-remitting MS, 11 with progressive-relapsing MS, 84 with secondary progressive MS, and 34 with primary progressive MS.

**Figure 1.**
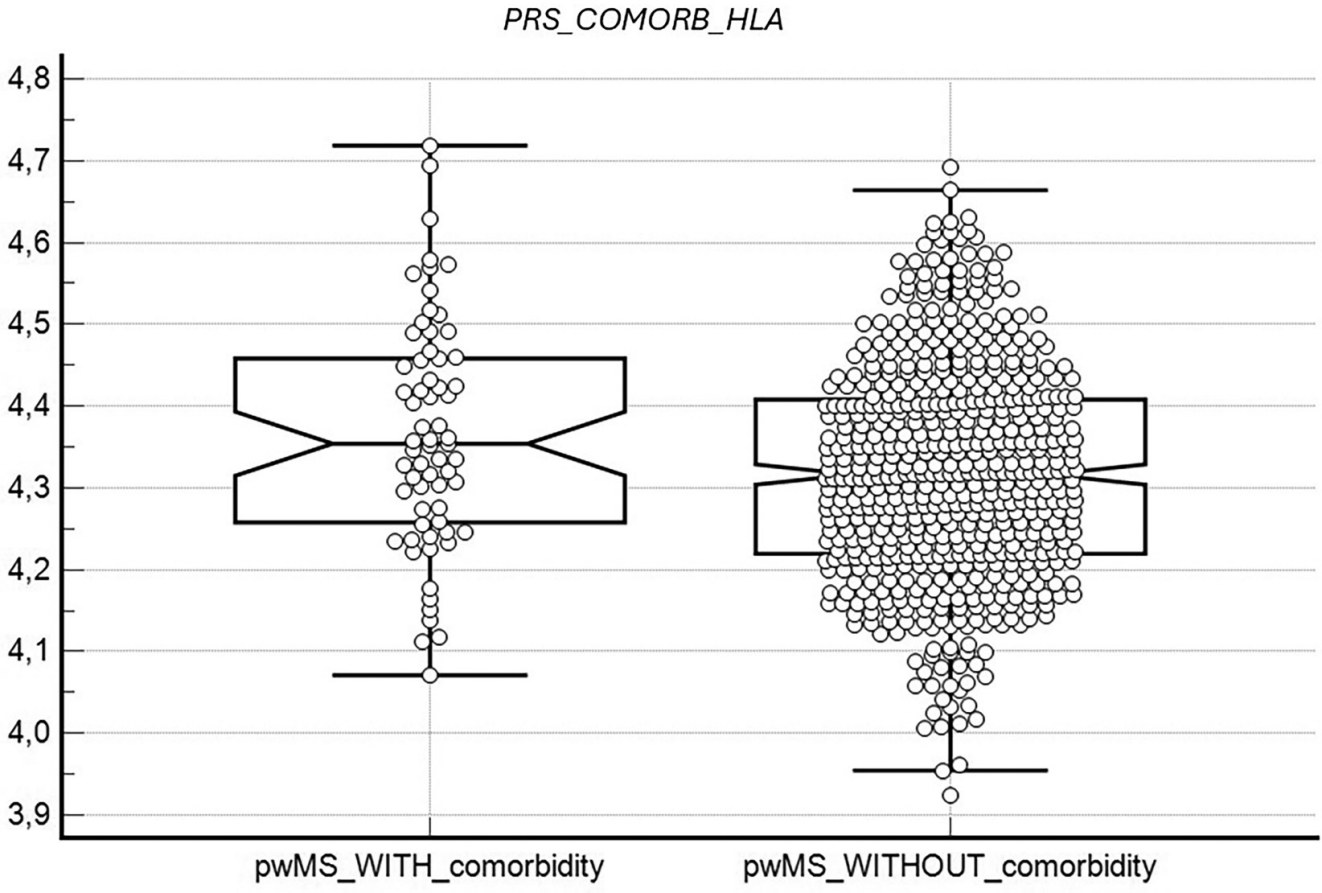
PRS_comorb_HLA distribution in pwMS with or without autoimmune comorbidities.

PRS: polygenic risk score; PwMS: persons with multiple sclerosis; HLA: human leukocyte antigen.

The mean cumulative *PRS_comorb_HLA* was found to be higher in pwMS with at least one additional AID compared to those without autoimmune comorbidities (Figure 1). This difference was statistically significant, as assessed by covariance analysis in models including age and the first three PCs (*p* = 0.0078) or age, sex, and first three PCs (*p* = 0.015) as covariates.

## Discussion

We interrogated public health archives to conduct a population-based analysis of the association with the presence of 14 AIDs in individuals with or without MS. Overall, we detected a significantly higher burden of autoimmune comorbidities among pwMS compared to those without MS.

Although the co-occurrence of MS with AIDs has been investigated in previous studies, including population-based analyses,^1,4,16^ this is the first study of its kind to examine rarer diseases like CIDP, sarcoidosis, and Behçet syndrome in a population-based setting. Furthermore, it is the first population-based study on autoimmune comorbidities in MS within an Italian population.

Our data demonstrate that all investigated AIDs are more frequent in pwMS compared to individuals without MS, with statistically significant differences observed for 11 of these conditions. These findings align with previous literature, which has consistently reported a higher overall prevalence of AIDs in pwMS compared to controls.^17–19^ In particular, prior population-based studies have shown that the prevalence of individual AIDs is generally equal to or higher in pwMS compared to controls, although variations exist between different populations and specific AIDs.^1,4,16^ Our study extends these findings by revealing a robust comorbidity between MS and less common AIDs that involve peripheral nerve demyelination, such as CIDP and Behçet syndrome. Although these conditions are rare, demyelination of peripheral nerves occurs in up to 5% of pwMS,^20^ and clinical evidence of CNS involvement has been documented in up to 8% of patients with CIDP^21^ and about 5% of patients with Behçet syndrome.^22^ This suggests that demyelination of both peripheral and central nerves may occur in specific subgroups of patients with MS or CIDP, whose characteristics remain poorly understood.

The robust association between MS and peripheral nerve demyelination may imply a shared pathophysiological mechanism. However, the rarity of these conditions means that our results may be influenced by their low prevalence, which calls for further validation in larger, more diverse populations. Of note, the link with CIDP has not been previously described in population-based studies, making it an important focus for future research given its potential clinical implications.

Altogether, these findings point towards a possible shared autoimmune pathogenesis or cross-reactivity between the peripheral and central nervous systems in predisposed individuals.

In good agreement with the largest population-based study investigating the incidence of 19 autoimmune diseases in 22 million individuals in the UK, our findings confirm the significant comorbidity with Hashimoto thyroiditis, myasthenia gravis, and SLE, while no significant comorbidity was found with AS. Conversely, the UK study did not find comorbidity with celiac disease or T1DM, whereas our analysis did not replicate the significant association with RA observed in the UK.^4^

These discrepancies may be due to differences in geographical, genetic, environmental, and ethnic compositions of the studied populations, as well as methodological differences, such as variations in data sources and case definitions used to retrieve cases from health information systems.

As a secondary objective, we investigated the genetic susceptibility to AID comorbidity in a random subset of pwMS with available genetic data. In particular, we evaluated the influence of known risk variants for MS and eight additional AIDs to test the hypothesis that the co-occurrence of two or more autoimmune conditions in the same patient may be triggered by common genetic susceptibility factors for autoimmunity. In this proof-of-concept study, we successfully linked genome data to routinely collected EHA, demonstrating the feasibility of using this methodology to examine health conditions, without imposing additional burden on the participants or relying on self-reported data, thereby overcoming recall bias. While the linkage of administrative data to identify clinical features and outcomes is well-established, the integration of EHA with biorepository and genetic data is novel and has rarely been applied to MS research.^23^

This secondary analysis was conducted on a relatively small-size subset, which limited our ability to stratify the results by specific comorbidities or analyze SNPs with sufficient statistical power. To address this limitation, we employed a cumulative PRS that considered known genetic variants associated with all nine AIDs investigated. Our results indicate a higher PRS in pwMS with comorbidities, suggesting that the genetic background may contribute to the co-occurrence of other AIDs in pwMS. Thus, MS individuals with a greater burden of genetic susceptibility factors for AIDs may be more likely to develop additional autoimmune conditions, although shared environmental exposures, such as smoking, could also play a role in the observed comorbidities.

To our knowledge, no other studies have assessed the relationship between genetic profiles and autoimmune comorbidities among pwMS identified using administrative data. However, while our results are promising, they are based on a small dataset and require further validation in larger, more representative sample sets. In addition, further studies are needed to identify other reasons for the co-occurrence of AID conditions beyond genetic factors. A recent large population-based study in the UK observed socioeconomic, seasonal, and regional disparities among several autoimmune disorders, suggesting the involvement of environmental factors in the pathogenesis of specific AIDs.^4^

In summary, the major strengths of our study include (i) using a large dataset of over 4 million individuals, which comprises all residents of Piedmont. This comprehensive dataset, collected through an administrative database search strategy, provided sufficient power to determine reliable epidemiological measures—even for rarer diseases—while minimizing the selection bias; (ii) employing routinely collected administrative data, which reduced recall bias compared to patient-based surveys. In addition, the integration of different data sources enabled us to capture all healthcare-related episodes across hospitals in Italy or regional services; (iii) conducting the study on a homogeneous, geographically defined population, which strengthened the generalizability of findings within this context; (iv) implementing a validated, highly reliable search algorithm for MS^9^ and the use of algorithms for other diseases that are widely employed in epidemiological research in Italy; and (v) developing a pipeline to link EHA with genetic data. In this regard, one challenge of linking genetic data with EHA is preserving participant privacy, according to the data-separation principle. In our study, all linked data were de-identified, and re-identification for genetic data was handled by clinicians who were blinded to the EHA identifiers, ensuring no access to personally identifiable information or linked administrative data.

We acknowledge several limitations of our study. First, the use of administrative data to identify comorbidities is subject to variability in data quality and completeness. Data quality can differ, and diagnoses may be inconsistently recorded due to differences in healthcare access and reporting practices. Nevertheless, the algorithm used to identify MS cases from EHA has been validated and demonstrated high sensitivity and specificity.^9^ Conversely, the algorithms for the other 14 AIDs, although having been used in previous epidemiologic studies, have not been validated, introducing a potential source of bias. Second, a potential surveillance bias exists due to differences in health system use between persons with and without MS. These differences may arise from varying disease severity, frequency of healthcare contacts, and unmeasured outpatient care. For example, individuals with more severe conditions are more likely to interact with the healthcare system, increasing their likelihood of being captured in EHA records, while undiagnosed individuals may remain undetected. We attempted to mitigate this bias by adjusting our estimates based on the number of hospitalizations. However, some of the conditions studied are largely managed without hospitalization. Ideally, we would have adjusted for the number of outpatient visits, but this was not possible due to limitations in the available administrative databases. Third, while other potential confounders (e.g. socioeconomic status) may exist, the intrinsic limitations of our available data sources precluded any adjustment for these variables. Fourth, we are aware of the possibility of having misclassified some individuals, especially pediatric cases, but this is unavoidable due to the anonymity of the data. Finally, the subset with available genetic data cannot be considered representative of the general Piedmont population, which limits the external validity of our findings.

In conclusion, our findings have several implications. Firstly, we have confirmed the association of MS with several AIDs, which may be due to shared genetic backgrounds. From a clinical standpoint, this strong association implies that individuals with either MS or an AID should be carefully monitored for possible coexisting chronic conditions, as these can considerably influence disease progression, treatment responses, and patient management. Secondly, our sub-study demonstrates the feasibility and reliability of linking genomic data with routinely collected administrative databases as a practical method to identify patient characteristics, such as the presence of comorbidities.

Overall, this approach lays the groundwork for future research into how genetic features influence other clinical characteristics, such as non-autoimmune comorbidity, and for a better understanding of the role of comorbidities in disease progression, as well as their potential impact on disease phenotype and brain damage.^24^ Given the exploratory nature of this work, further validation of our findings in larger and more representative sample sets is clearly needed.

## Supplemental Material

sj-pdf-1-mso-10.1177_20552173251349671 - Supplemental material for Association of autoimmune comorbidities in persons with multiple sclerosis from a population-based study with genetic linkage Supplemental material, sj-pdf-1-mso-10.1177_20552173251349671 for Association of autoimmune comorbidities in persons with multiple sclerosis from a population-based study with genetic linkage by Roberto Gnavi, Nadia Barizzone, Roberta Picariello, Paolo Emilio Alboini, Nicola Pomella, Muralidharan Thavamani, Martina Tosi, Endri Visha, Valentina Ciampana, Domizia Vecchio, Paola Cavalla, Maurizio Leone and Sandra D'Alfonso in Multiple Sclerosis Journal – Experimental, Translational and Clinical

sj-pdf-2-mso-10.1177_20552173251349671 - Supplemental material for Association of autoimmune comorbidities in persons with multiple sclerosis from a population-based study with genetic linkage Supplemental material, sj-pdf-2-mso-10.1177_20552173251349671 for Association of autoimmune comorbidities in persons with multiple sclerosis from a population-based study with genetic linkage by Roberto Gnavi, Nadia Barizzone, Roberta Picariello, Paolo Emilio Alboini, Nicola Pomella, Muralidharan Thavamani, Martina Tosi, Endri Visha, Valentina Ciampana, Domizia Vecchio, Paola Cavalla, Maurizio Leone and Sandra D'Alfonso in Multiple Sclerosis Journal – Experimental, Translational and Clinical

sj-pdf-3-mso-10.1177_20552173251349671 - Supplemental material for Association of autoimmune comorbidities in persons with multiple sclerosis from a population-based study with genetic linkage Supplemental material, sj-pdf-3-mso-10.1177_20552173251349671 for Association of autoimmune comorbidities in persons with multiple sclerosis from a population-based study with genetic linkage by Roberto Gnavi, Nadia Barizzone, Roberta Picariello, Paolo Emilio Alboini, Nicola Pomella, Muralidharan Thavamani, Martina Tosi, Endri Visha, Valentina Ciampana, Domizia Vecchio, Paola Cavalla, Maurizio Leone and Sandra D'Alfonso in Multiple Sclerosis Journal – Experimental, Translational and Clinical

sj-pdf-4-mso-10.1177_20552173251349671 - Supplemental material for Association of autoimmune comorbidities in persons with multiple sclerosis from a population-based study with genetic linkage Supplemental material, sj-pdf-4-mso-10.1177_20552173251349671 for Association of autoimmune comorbidities in persons with multiple sclerosis from a population-based study with genetic linkage by Roberto Gnavi, Nadia Barizzone, Roberta Picariello, Paolo Emilio Alboini, Nicola Pomella, Muralidharan Thavamani, Martina Tosi, Endri Visha, Valentina Ciampana, Domizia Vecchio, Paola Cavalla, Maurizio Leone and Sandra D'Alfonso in Multiple Sclerosis Journal – Experimental, Translational and Clinical
