# Supplementary material for: Association of autoimmune comorbidities in persons with multiple sclerosis from a population-based study with genetic linkage
Source: Mult Scler J Exp Transl Clin. 2025 Jul 3;11(3):20552173251349671. doi: 10.1177/20552173251349671 (PMC12227931; doi:10.1177/20552173251349671)
Supplement: sj-pdf-1-mso-10.1177_20552173251349671 - Supplemental material for Association of autoimmune comorbidities in persons with multiple sclerosis from a population-based study with genetic linkage [file sj-pdf-1-mso-10.1177_20552173251349671.pdf]

**Supplementary Table S1.** Criteria used to identify subjects with autoimmune diseases

| **Disease** | **Exemption from co-payment code** | **Drug prescriptions [1] (ATC codes)** | **Hospital discharge diagnosis [2] (ICD9-CM codes)** |
|---|---|---|---|
| Rheumatoid arthritis[3] | 006 | L01BA01,A07EC01, H02AB07,H02AB04, L01XC02,L04AA24, L04AB01,L04AB02, L04AB04,L04AB05, L04AB06, L04AC03, L04AC07, L04AC14, L04AA29, L04AA37, L04AX01,L04AD01 | 714.0 |
| Celiac disease | 059 or RI0060 | | 579.0 |
| Chronic inflammatory demyelinating polyneuropathy | RF0180 | | 357.81 |
| Type 1 diabetes | Regional Register of Diabetes | A10A without any prescription of A10B, and ≤35 years | 250x1 or 250x3 |
| Hashimoto thyroiditis | 056 | | 245.2 |
| Hypothyroidism | | H03AA01, H03AA02, H03AA03 | Excluding thyroidectomy (partial or total) or $I^{131}$irradiation in the previous 5 years (062-064 or 92.28) |
| IBD[4] | 009 or 031 | | 555 or 556, (excluding 5564 or 5568) |
| Systemic lupus erythematosus | 028 | | 710.0 |
| Myasthenia gravis | 034 | N07AA02 | 358.0 |
| Psoriatic arthritis | 045 | | 696.0 |
| Sarcoidosis | RH0011 or RA0041 | | 135 |
| Behçet syndrome | RC0210 | | 136.1 |
| Guillain-Barré syndrome | RF0183 RFG101 | | 357.0 |
| Sjogren's syndrome | 030 | | 710.2 |
| Ankylosing spondylitis | 054 | | 720.0 |

A subject was considered affected by a disease if at least one of the conditions indicated in the table was met.

[1] At least two prescriptions in two different dates in 2017.

[2] At least one hospitalization from 2013 to 2017; primary or secondary diagnosis at discharge.

[3] To confirm the diagnosis of AR a consultation with a rheumatologist, in addition to drug prescriptions, was requested (data from the regional outpatient database) in 2017.

[4] IBD was excluded from final linkage analysis, due to an error in the coding of IBD in the exemption from co-payment archives, which prevented its use.
