# Supplementary material for: Association of autoimmune comorbidities in persons with multiple sclerosis from a population-based study with genetic linkage
Source: Mult Scler J Exp Transl Clin. 2025 Jul 3;11(3):20552173251349671. doi: 10.1177/20552173251349671 (PMC12227931; doi:10.1177/20552173251349671)
Supplement: sj-pdf-2-mso-10.1177_20552173251349671 - Supplemental material for Association of autoimmune comorbidities in persons with multiple sclerosis from a population-based study with genetic linkage [file sj-pdf-2-mso-10.1177_20552173251349671.pdf]

Supplementary Table S2. Number of non-HLA SNPs used to construct each disease-specific PRSs and GWAS used as a source study for each disease.

DISEASE	N SNPs	GWAS
Rheumatoid arthritis	91	Okada 2014
Celiac disease	71	Ricaño-Ponce 2020, Van Heel 2007, Trynka 2011.
Type 1 diabetes	31	Sharp 2019
Hashimoto thyroiditis	39	Brčić 2019, Tomer 2015, Cooper 2012, Sakaue 2021
Hypothyroidism	139	Kichaev 2019, Sakaue 2021, Pickrell 2016, Eriksson 2012, Teumer 2018, Denny 2011
Systemic lupus erythematosus	49	Julià 2018
Psoriasis	54	Tsoi 2017
Ankylosing spondylitis	23	IGAS 2013
Multiple sclerosis	201	IMSGC 2019

References:

Okada Y, Wu D, Trynka G et al. Genetics of rheumatoid arthritis contributes to biology and drug discovery. Nature 2014; 506(7488):376-81. supplementary table 1

Ricaño-Ponce I, Gutierrez-Achury J, Costa AF, et al. Immunochip meta-analysis in European and Argentinian populations identifies two novel genetic loci associated with celiac disease. Eur J Hum Genet 2020; 28(3):313-323.

Van Heel DA, Franke L, Hunt KA, et al. A genome-wide association study for celiac disease identifies risk variants in the region harboring IL2 and IL21. Nat Genet 2007; 39(7):827-9.

Trynka G, Hunt KA, Bockett NA, et al. Dense genotyping identifies and localizes multiple common and rare variant association signals in celiac disease. Nat Genet 2011; 43(12):1193-201.

Sharp SA, Rich SS, Wood AR, et al. Development and Standardization of an Improved Type 1 Diabetes Genetic Risk Score for Use in Newborn Screening and Incident Diagnosis. Diabetes Care 2019; 42:200–207. Supplementary table S5: Thirty-two SNPs marking 31 non-HLA loci used in the GRS2

Brčić L, Barić A, Gračan S, et al. Genome-wide association analysis suggests novel loci underlying thyroid antibodies in Hashimoto's thyroiditis. Sci Rep. 2019;9(1):5360.

Tomer Y, Dolan LM, Kahaly G, et al. Genome wide identification of new genes and pathways in patients with both autoimmune thyroiditis and type 1 diabetes. J Autoimmun. 2015;60:32-9.

Cooper JD, Simmonds MJ, Walker NM, et al. Seven newly identified loci for autoimmune thyroid disease. Hum Mol Genet. 2012;21(23):5202-8.

Sakaue S, Kanai M, Tanigawa Y. A cross-population atlas of genetic associations for 220 human phenotypes. Nat Genet. 2021;53(10):1415-1424.

Kichaev G, Bhatia G, Loh PR, et al. (2019) Leveraging Polygenic Functional Enrichment to Improve GWAS Power. Annals Journal Human Genetics 104(1):65-75.

Pickrell JK, Berisa T, Liu JZ, Ségurel L, Tung JY, Hinds DA. (2016) Detection and interpretation of shared genetic influences on 42 human traits [published correction appears in Nat Genet. 2016 Sep 28;48(10):1296]. Nature Genetics 48(7):709-717.

Eriksson N, Tung JY, Kiefer AK, et al. (2012) Novel associations for hypothyroidism include known autoimmune risk loci. PLoS One. 7(4):e34442.

Teumer A, Chaker L, Groeneweg S, et al. (2018) Genome-wide analyses identify a role for SLC17A4 and AADAT in thyroid hormone regulation. Nature Community 2018;9(1):4455. Published 2018 Oct 26.

Denny JC, Crawford DC, Ritchie MD, et al. (2011) Variants near FOXE1 are associated with hypothyroidism and other thyroid conditions: using electronic medical records for genome- and phenome-wide studies. Annal Journal of Human Genetics 89(4):529-542.

Julià A, López-Longo FJ, Pérez Venegas JJ, et al. Genome-wide association study meta-analysis identifies five new loci for systemic lupus erythematosus. Arthritis Res Ther. 2018; 20:100. Supplementary table 3 (Association results for the 52 previously known SLE risk loci)

Tsoi LC, Stuart PE, Tian C et al. Large scale meta-analysis characterizes genetic architecture for common psoriasis associated variants. Nat commun 2017; 8:15382.

International Genetics of Ankylosing Spondylitis Consortium (IGAS), Cortes A, Hadler j et al. Identification of multiple risk variants for ankylosing spondylitis through high-density genotyping of immune-related loci. Nat Genet. 2013; 45(7):730-8 (Table 1. Non-MHc associations with ankylosing spondylitis susceptibility.)

International Multiple Sclerosis Genetics Consortium (IMSGC). Multiple sclerosis genomic map implicates peripheral immune cells and microglia in susceptibility. Science. 2019; 365(6460):eaav7188.
