# Supplementary material for: Association of autoimmune comorbidities in persons with multiple sclerosis from a population-based study with genetic linkage
Source: Mult Scler J Exp Transl Clin. 2025 Jul 3;11(3):20552173251349671. doi: 10.1177/20552173251349671 (PMC12227931; doi:10.1177/20552173251349671)
Supplement: sj-pdf-3-mso-10.1177_20552173251349671 - Supplemental material for Association of autoimmune comorbidities in persons with multiple sclerosis from a population-based study with genetic linkage [file sj-pdf-3-mso-10.1177_20552173251349671.pdf]

Supplementary Table S3. HLA variants used for the disease specific PRSs

**DISEASE**	**HLA variant used**	**weight**
Rheumatoid arthritis	HLA_DRB1*04:04	3
Celiac disease	HLA:DQB1*02:01	3
Type 1 diabetes	HLA_DRB1*03:01	3
Hashimoto thyroiditis	HLA_DRB1*03:01	3
Hypothyroidism	6:31018407:A:G<br>6:32586794:T:G<br>6:33048538:T:C<br>6:35537964:C:T	1<br>1<br>1<br>1
Systemic lupus erythematosus	HLA_DRB1*03:01	3
Psoriasis	HLA_C*06:02	3
Ankylosing spondylitis	HLA_B*27:05	3
Multiple sclerosis	*HLA-DRB1*15:01*	*3*

For each HLA allele, we determined the number of allele frequency in each PWMS (0, 1, 2) and multiplied it for the reported weight. Compared to HLA alleles, all non-HLA variants have a weight = 1.
