# Supplementary material for: Association of autoimmune comorbidities in persons with multiple sclerosis from a population-based study with genetic linkage
Source: Mult Scler J Exp Transl Clin. 2025 Jul 3;11(3):20552173251349671. doi: 10.1177/20552173251349671 (PMC12227931; doi:10.1177/20552173251349671)
Supplement: sj-pdf-4-mso-10.1177_20552173251349671 - Supplemental material for Association of autoimmune comorbidities in persons with multiple sclerosis from a population-based study with genetic linkage [file sj-pdf-4-mso-10.1177_20552173251349671.pdf]

Supplementary Figure S1. Distribution PCs 1-3 in the population used for the genetic analysis

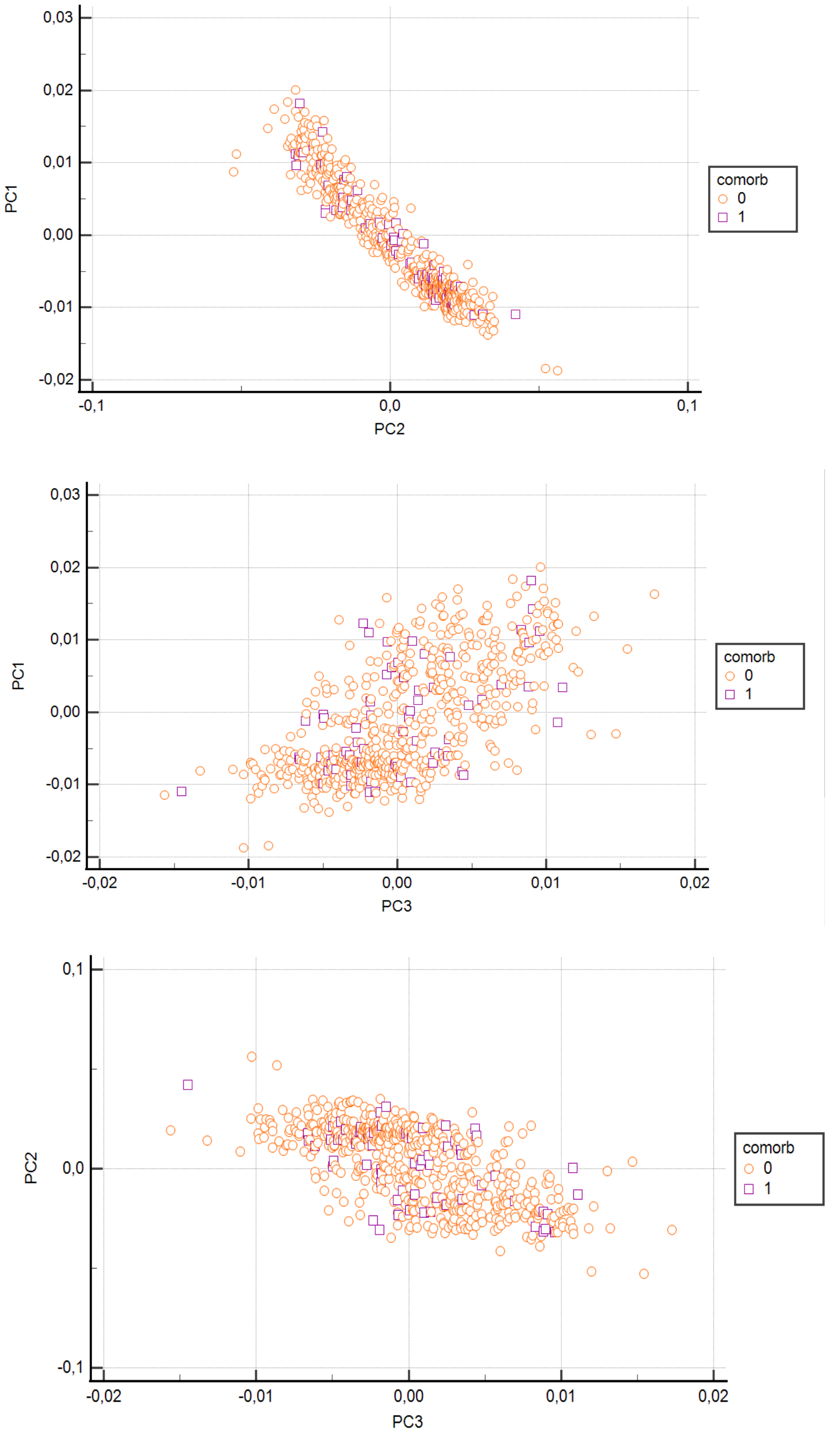

Each dot is an individual, classified basing on the comorbidity status: 1= pwMS with AIDs comorbidity, 0= pwMS without comorbidity.
